# The Spatial Distribution of Renal Fibrosis Investigated by Micro-probe Terahertz Spectroscopy System

**DOI:** 10.3390/diagnostics12071602

**Published:** 2022-06-30

**Authors:** Han Li, Jiarong Ding, Huan Zhang, Maoting Li, Xueli Lai

**Affiliations:** 1 The First Rehabilitation Hospital of Shanghai, School of Medicine, Tongji University, Shanghai 200092, China; 1805163@tongji.edu.cn; 2 Shanghai Institute of Intelligent Science and Technology, Tongji University, Shanghai 200092, China; 3 Shanghai Changhai Hospital, Navy Medical University, Shanghai 200433, China; djr567@163.com (J.D.); braadcate@163.com (H.Z.)

**Keywords:** microprobe terahertz spectroscopy, renal fibrosis, non-invasive, early diagnosis

## Abstract

Renal fibrosis, which is characterized as progressive extracellular matrix accumulation, is a common feature of different stages of chronic kidney disease, and the degree of fibrosis is strongly associated with renal function. In clinical practice, precise understanding of the space distribution of fibrosis is extremely important for the diagnosis and prognosis of renal disease. Rapid advances in terahertz (THz) technology have been made, and this technology has a broad application in bio-detection, as it can interact and measure the collective vibrations and rotations of molecular groups. It is well known that hydroxyproline (HYP) is the key component of collagen, which is synthesized by fibroblasts to maintain the extracellular matrix, and HYP content detection in tissue homogenate can be achieved by classical biochemistry method. In this study, a THz microprobe system was employed to conduct THz microspatial scanning with a resolution of 20 µm. Both the content and distribution of HYP were directly characterized by the THz absorption spectrum. The absorption intensity in the THz spectrum was used to determine HYP density in renal tissues; therefore, the fibrosis change in the kidneys can be determined using THz scanning at micrometer resolution, which provides more possibilities for precise diagnosis of renal fibrosis.

## 1. Introduction

Based on the data of the 2012 National Epidemiological Investigation [1], the prevalence of chronic kidney disease (CKD) in China is approximately 10.8%, which is estimated at around 130 million CKD cases. In addition, renal fibrosis is an important pathological marker during renal function decline. Renal fibrosis is a chronic progressive fibrotic nephropathy, and is the eventual outcome of almost all CKDs and progressive nephropathy [2]. A comparable proportion of the population with renal fibrosis eventually progresses to advanced renal failure and requires life-long dialysis or kidney transplantation, resulting in an immense burden on patients, families, and society [3]. The primary pathological presentations of renal fibrosis are inflammatory cell infiltration, fibroblast activation and proliferation, accumulation of large amounts of extracellular matrix (ECM), and loss of renal intrinsic cells [4,5]. Treatment of renal fibrosis is extremely difficult, and early diagnosis of this disease has been a focus and a challenge for nephrologists to overcome.

The ideal diagnosis of renal fibrosis should be precise and non-invasive. At present, diagnosis of renal fibrosis is mainly dependent on histological and radiologic assessment, as well as biomarker detection. Histology is currently the gold standard for the clinical diagnosis of renal fibrosis, as histological images provide direct evidence of fibrosis severity and precise subcellular change, by specific staining. Nevertheless, all of these changes only reflect the pathological features in a tissue slice, rather than the overall renal tissue changes. Additionally, the risk of renal hemorrhage in the aging, obese, and anticoagulant-using population limits the conduction of kidney biopsies [6]. Radiologic methods include ultrasound, magnetic resonance imaging (MRI), and optical coherence tomography (CT), which are non-invasive techniques. Physicians can determine whether fibrosis has occurred in the patient by observing kidney elasticity, oxygen content, and blood perfusion. According to the image post-processing, the 2D or 3D renal fibrosis status in computerized tomography or MRI, could be acquired under about 1 mm resolution. However, renal fibrosis identification tends to be affected by factors such as blood pressure, weight, respiratory movements, and differences in subjective judgments between observers, resulting in low precision [7]. Generally, radiological images are indirect findings, which reflect the physical or chemical status of fibrotic tissue. The specific staining of biomacromolecules in tissue could be the evidence of early-stage fibrosis. In the latest research, some biological molecules showed the potential ability to illustrate fibrosis. For example, urinary microRNA 214 (miR-214) can be used as a biomarker to monitor early renal fibrosis, but its sensitivity and specificity remain to be determined [8]. 

In summary, evaluation of the proportion, degree, and spatial distribution of renal fibrosis is extremely important for understanding kidney disease conditions. There is an urgent need to develop a non-invasive strategy that integrates biomacromolecule labeling and spatial distribution to achieve an early diagnosis of renal fibrosis. 

In recent years, terahertz (THz) science and technology has demonstrated great potential in medical diagnoses. THz (1 THz = 10^12^ Hz) refers to a frequency of 100 gigahertz (GHz) to 10 THz, and the corresponding wavelength is from 3 mm to 30 µm, which is a broad electromagnetic wave spectral region between millimeter waves and infrared [9,10,11,12,13,14]. THz technology has importance in life sciences. In addition to conventional spectral measurement information, THz technology can also provide low-frequency vibration, hydrogen bond stretching, and bond rotation information for liquids and gases [15]. Currently, THz technology is non-destructive, accurate, and fast, and can provide good penetration [16]. Hence, THz spectroscopy may be an effective method for diagnosing various diseases [17]. A previous study established a test standard for using the THz spectrum in rodent tissue samples [18]. THz spectroscopic technique has been proven to have an effect on the detection of skin, breast, tongue, liver, and colon tumors [19,20,21]. The THz spectrum and concentration gradient were constructed by biomarker screening and by testing pure standard samples. After excluding individual differences by interference, the evaluation was confirmed, and the effective characteristic peaks were retained. Finally, THz spectroscopy was shown to play an important role in the rapid, accurate, and early diagnosis of diseases, which resulted in further exploration to achieve rapid, accurate, low-cost, early, precise, and qualitative diagnoses of diseased tissues using the THz spectrum.

For the purpose of renal fibrosis early diagnosis, biomacromolecule detection is the most promising approach to explore. High levels of hydroxyproline (HYP) are well-established as the feature of active fibroblast cells, thus, Peng et al. started the rodent research to observe whether HYP can be recognized by the THz system, instead of the biochemistry method [18], after confirming that HYP had a clear THz characteristic peak at ~1.2 THz that changes almost linearly with concentration. Although this is an extremely useful experiment to quantitatively identify the concentration of HYP, which is closely related to renal fibrosis, it still cannot demonstrate the spatial distribution of HYP and renal fibrosis. This is because the terahertz wavelength was about 300 µm, the spatial resolution of which is too poor for the spatial distribution measurement (around ~mm). However, knowledge of the spatial distribution of HYP, together with the location of fibrosis, is extremely important for clinicians to judge the severity of fibrosis, so that more reasonable therapies can be provided to patients according to the spatial distribution of HYP, together with the location of fibrosis.

In the present study, a THz microprobe system was employed to conduct THz spectrum testing of kidney tissues in mice with chronic renal fibrosis. THz spatial scanning with a resolution of 20 µm was achieved, resulting in direct observation of the THz imaging spectrum to determine the spatial distribution of renal fibrosis. As renal fibrosis and HYP have a direct relationship [18], and the HYP content can be directly characterized by THz absorption peaks. Therefore, here, terahertz time-domain spectroscopy (THz-TDS) with micrometer resolution was employed to determine not only the density of HYP, but also the location of lesions in renal tissues, using the THz beam. Compared with the complex preparation time of pathological specimens, THz spectral detection can achieve rapid and high-resolution imaging of the spatial distribution of fibrotic tissues, provide more favorable evidence for evaluating mouse renal function and prognosis, and also provide more thorough exploration in biological tissue specimens. We hope that this proposed method can provide more possibilities for the precise diagnosis of renal fibrosis. However, due to the limitation of the energy of the THz beam, the entire experiment was conducted in vitro,. In the future, THz spectrum testing may be a promising way for clinicians to make the decision of diagnosis and treatment in vivo.

## 2. Materials and Methods

### 2.1. Micro-probe Terahertz Time-Domain Spectroscopy (Microprobe THz-TDS)

**Figure 1:**
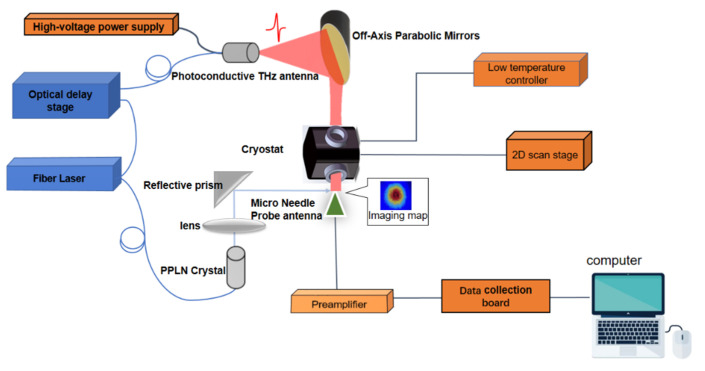
The schematic of Microprobe THz time-domain spectroscopy.

Microprobe THz-TDS equipment was used in this study which is illustraed in Figure 1. In the experiment, a 1560-nm fs laser was used, with a pulse duration of 100 fs, repetition rate of 80 MHz, and mean power of 150 mW. The laser ray emitted was divided by a 1/4 beam splitter into a pump beam and a probe beam. The pump beam was guided onto an indium gallium arsenide emitter through an optical fiber. The THz electromagnetic wave emitted by the photoconductive antenna was made parallel by the parabolic mirror and directed onto the sample. The probe beam underwent frequency multiplication by periodically poled lithium niobate (PPLN) to produce a 780-nm laser (approximately 30 mW) and was directed onto the probe photoconductive antenna (gallium arsenide) to measure the THz signals that penetrated the sample. In the experiment, the effective bandwidth of the signal was 0.2 to 2.0 THz, the spectral resolution was approximately 15 GHz, and the spatial resolution reached 20 µm. The spectrum was averaged 32 times to ensure a high signal-to-noise ratio. The THz pulse waveform acquisition speed was 10 scans/s.

### 2.2. Thermostatic Liquid Nitrogen Cryostat

A variable thermostatic liquid nitrogen cryostat (OptistatDN2, Oxford Instruments) with high-density polyethylene glass, a commercial product designed for spectroscopy, was used. The temperature of the cryostat was able to continuously vary between 77 K to 500 K with a precision of ±0.5 K. In the experiment, the cryostat was placed between the THz focal points, which were between the off-axis parabolic mirrors in the THz-TDS. The sample was placed in the cryostat, and THz radiation was passed through the sample to obtain a fingerprint spectrum.

### 2.3. Preparation of Animal Model Samples

Eight-to-nine-week-old male Institute of Cancer Research (ICR) mice weighing 32–35 g were provided by the Tongji University Laboratory Animals Center, and were bred and maintained according to the Guide for the Care and Use of Laboratory Animals published by the US National Institutes of Health. The Laboratory Animal Ethics Committee of the Navy Medical University approved the use of a unilateral ureteral obstruction (UUO) mouse model to study renal fibrosis. A total of 18 male ICR mice were randomized into a normal (sham surgery) group (*n* = 9) and renal fibrosis (UUO surgery) group (*n* = 9). Sodium pentobarbital (3%, 2 mL/kg) was administered by intraperitoneal injection for anesthesia. After anesthesia, on the right kidney, UUO or sham surgery was performed. The right ureter was ligated with 6–0 surgical suturesk, and sham-operated mice underwent the same surgery without ligation of one ureter. Mice were euthanized 14 days after surgery. Right kidneys were harvested from mice in the UUO and normal groups. The resected renal tissues were cut into 600-µm-thick sections that were rapidly freeze-dried using liquid nitrogen. The freeze-dried renal tissues were placed in the THz probe system for imaging.

### 2.4. Hematoxylin and Eosin and Masson’s Staining

Kidney tissue was fixed in 4% paraformaldehyde for 24 h. Then, the kidney tissues were paraffin-embedded according to the standard protocol. Finally, the paraffin blocks were sectioned into 4-μm-thick slices. Both hematoxylin and eosin (H&E) and Masson’s staining were conducted, and the slices were subjected to a microscopic histological examination.

## 3. Results

### 3.1. Pathological Findings Showed Renal Tubulointerstitial Fibrosis (RIF)

**Figure 2:**
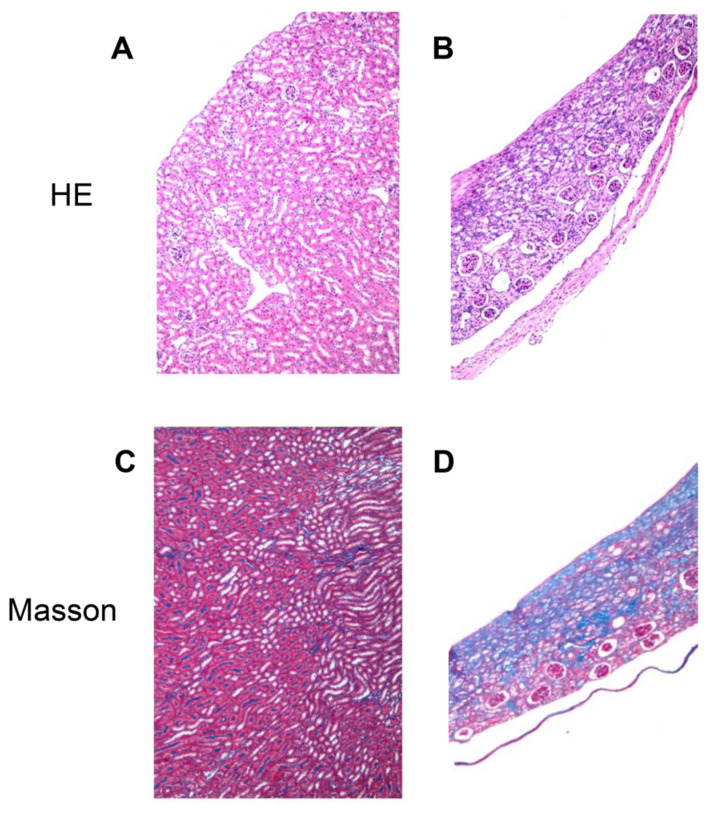
Renal pathological finding in mice underwent sham surgery and unilateral ureteral obstruction (UUO). (**A**) No obvious damage could be found in normal kidney slice. (**B**) Glomerulosclerosis, tubular atrophy, parenchymal loss, and inflammatory cells infiltration were significant in H&E staining of renal fibrosis section. (**C**,**D**) Masson’s staining indicated small amount collagen deposition (blue staining) in normal kidney section, as well as strong tubulointerstitial collagen staining in renal fibrosis mice.

The pathological images of renal tissues from renal fibrosis and normal groups are shown in Figure 2. H&E staining indicated the worse parenchymal loss, tubular atrophy, and more significant infiltration of inflammatory cells in renal fibrosis slices (Figure 2B) than that in normal renal tissue (Figure 2A). Compared with sham surgery mice (Figure 2C), Masson’s staining also revealed that UUO mice showed fibrotic renal tissue with more extensive collagen deposition (Figure 2D). The positive blue staining in the mouse kidney demonstrated that the collagen fiber was synthesized. However, the aforementioned changes in the slices only occurred during the middle–late phase of fibrosis. These characteristics suggest that fibrosis occurred in the renal tissues.

### 3.2. THz-TDS Imaging

**Figure 3:**
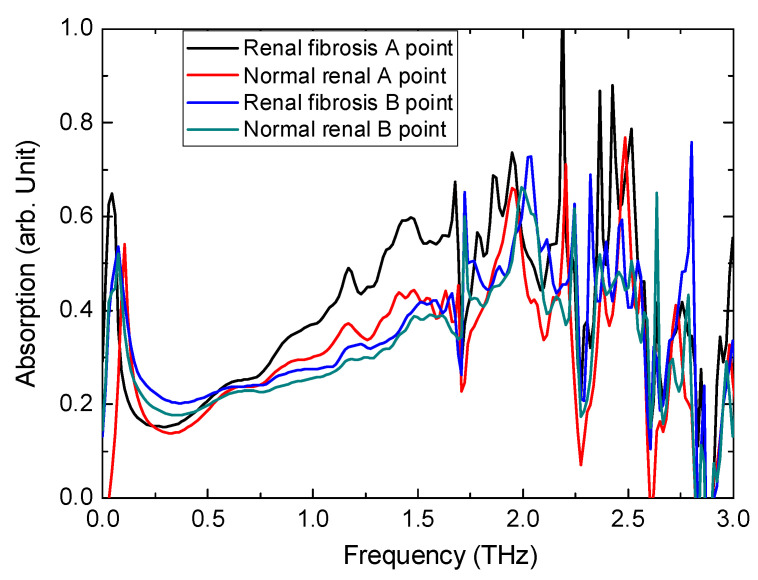
THz absorption spectra of mouse renal tissues in normal and renal fibrosis group. Black line is the absorption spectrum of the renal fibrosis tissue from the renal fibrosis group (center point). Blue line is the absorption spectrum of the renal fibrosis tissue from the renal fibrosis group (edge point). Red line is the absorption spectrum of the normal renal tissue from the normal group (center point). Green line is the absorption spectrum of the normal renal tissue from the normal group (edge point).

A previous study confirmed that a visible THz characteristic peak exists for HYP, and linear changes occur as the concentration changes [18]. This result indicates that the whole THz signal in the time-domain decreases when the HYP concentration increases (absorption caused by HYP). Based on this finding, THz waves were used to detect the HYP in mouse renal tissue sections. Figure 3 shows the THz absorption spectrum of renal tissues from mice from the UUO surgery and sham groups. The absorption curves were obtained from the Fourier transformation of time-domain signals. Then, using the function *I* = *I*_0_exp (−*α* *×* *l*), where *I* is the intensity of the output THz wave through the tissue sample, *I*_0_ is the intensity of the entrance THz wave, and *l* is the thickness of the tissue sample, the absorption coefficient *α* can be obtained as an arbitrary (arb.) unit. As shown before [18], L-HYP had a clear THz characteristic peak at ~1.2 THz that can represent the concentration of the HYP. Here, the microprobe THz system also showed the same results, i.e., that the HYP was concentrated in fibrotic renal tissues, and the concentration of HYP can be represented by the intensity of the absorption coefficient. This figure shows that the absorption of the entire spectrum was higher, together with a clear peak at 1.2 THz for renal fibrosis tissue, especially for the A point (center point), and almost no absorption signal was found at the edge B point. These results prove that the HYP gathers in the center of renal tissue. On the other hand, for normal tissue, these HYP absorption peaks, i.e., at 1.2 THz, were very low, which proves that concentrations of HYP is normal renal tissue is low, as well as means mild fibrosis.

**Figure 4:**
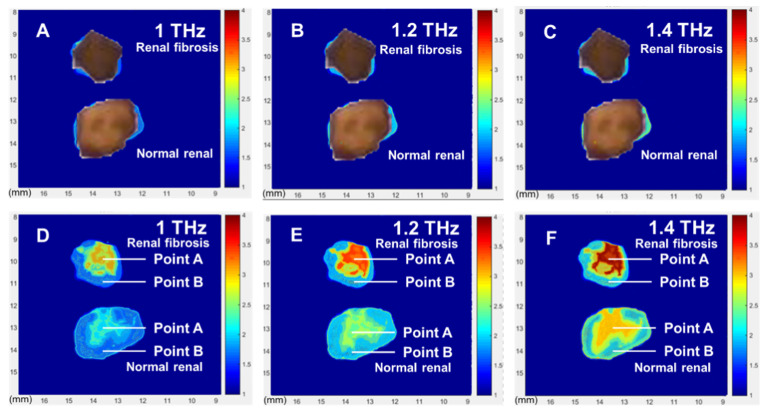
Spatial distribution of HYP in terahertz absorption images. (**A**–**C**) are the optical images from the THz imaging of the renal fibrosis tissues from UUO mice and normal tissues from the sham group, corresponding to pictures (**D**–**F**). (**D**–**F**) are the THz images of these tissues at the frequencies of 1.0, 1.2, 1.4 THz, respectively. The pictures above are of the renal fibrosis tissues, and the pictures below are of the normal renal tissues.

Figure 3 shows the absorption spectra of specified points. Then, using scanning technology, we can obtain the absorption spectra of the whole tissues. Here, we do not move the terahertz beam, due to the complicated optical path. Using a Micro Servo Motor, we move the sample to get a new data, together with the thermostatic liquid nitrogen cryostat. In Figure 4, from pictures D to F, are the terahertz images of the absorption of renal fibrosis tissues from UUO mice and normal tissues from the sham group under different frequencies. As a comparison, the pictures A–C (Figure 4) show optical images covered on the THz imaging of these tissues, corresponding to pictures D to F. The HYP distribution in the 600-µm-thick pathological tissue sections of the renal can be used to clearly distinguish the distribution of fibrotic sites. We can clearly see that: (1) for the renal fibrosis tissue, the absorption in the center of the tissue is much higher than that at the edge, which is the same result as shown in Figure 3. This clearly proves that the HYP gathers in the center of the tissue, and that the fibrosis is also much more serious in the center. (2) if we compare the results between the center-of-fibrosis tissue and normal tissue, we find that the absorption difference is very large, especially at 1.2 THz and 1.4 THz; this is because HYP has a very strong absorption peak at ~1.2 THz and ~1.4 THz, which mainly exists in the center-of-fibrosis tissue. Using this method, routine pathological staining is not required, and the spatial distribution of HYP-containing proteins can be determined.

## 4. Discussion

Renal fibrosis is a complex and dynamic process. During disease onset, scar tissue replaces functional tissue, changing the physiological function of the organ and resulting in failure. In addition, the structure of the kidneys becomes disorderly, damage to the glomeruli and interstitium occurs, and there is an imbalance in the synthesis and degradation of ECM components, thereby leading to aberrant increases in ECM in tissues and organs, and excessive deposition [4,5]. Generally, during the early stages of organ fibrosis, proline will be hydroxylated by prolyl hydroxylase into HYP; this is because the HYP is a characteristic of collagen, which is not widespread in other kinds of protein. Thus, the HYP content in tissues represents the degree of collagen metabolism. As collagen is a major component of the ECM, the HYP content also reflects the degree of tissue fibrosis [6,7,18]. However, none of experiments were able to reach the microresolution level. The current study found that THz signals that characterize the HYP distribution were highly expressed in fibrotic renal tissues, and had high concordance with traditional pathology tests. By observing Masson-stained renal tissue sections, this study found that widespread collagen hyperplasia was present in renal tissues in UUO model mice. Microprobe THz-TDS has the ability to solve this problem, which could be used for effective identification of tissue sites with biological macromolecules, such as HYP, and to capture some organ-specific biological macromolecules; therefore, Microprobe THz-TDS can indicate the location and severity of lesions, and aid clinicians in making accurate judgments of disease severity.

THz waves are extremely sensitive to water content in biological tissues, which conceals the minor changes in chemical substances. For a long time, differences in tissue water content were considered to be the main reason for differences in THz imaging signals, because water shows strong THz absorption [17,22,23]. However, THz waves can also detect the slight changes in tissues after samples have undergone dehydration or paraffin embedding, suggesting that THz imaging can be used to distinguish structures or ingredients of different pathological tissues. In addition, Microprobe THz waves have a higher spatial resolution than other THz imaging methods, and can also penetrate the epidermis. Furthermore, the use of time-domain imaging can allow the conversion of amplitude and phase information of samples into three-dimensional (3D) images. These characteristics enable THz waves to be used for in vitro tissue imaging and in vivo imaging for some superficial tissues. This characteristic expands the application of Microprobe THz-TDS in medicine, i.e., Microprobe THz-TDS can be used to detect the distribution sites of HYP and other biological macromolecules in superficial fibrotic diseases, such as dermatomyositis, scleroderma, and systemic sclerosis, and it can enable clinicians to determine the severity and specific locations of these diseases in a non-invasive manner in the near future; at present, this process is still performed in vitro.

The advantages of using this sample preparation method are as follows: (1) the sample is freeze-dried, and therefore, strong THz absorption by water is avoided; (2) the samples never deteriorate, and the temperature is 77 K. However, this probe system uses multiple lenses (including the cold cavity lens); therefore, the effective frequency spectral range is only 1.5 THz, and is further decreased to 0.8 THz with samples, and the 1.02 THz peak cannot be observed.

In a previous study, a microprobe THz system was employed to measure the spatial distribution of HYP, which is closely related to the location of renal fibrosis. THz spatial scanning with a resolution of 20 µm was achieved, The current study was based on this previous study, and employed Microprobe THz-TDS to directly resolve the spatial resolution of the THz spectrum of renal fibrosis tissues using 600-μm tissue sections. The spatial distribution of HYP was observed to determine the precise spatial localization of fibrotic tissues in the entire kidney. The use of Microprobe THz-TDS could effectively avoid the limitations of currently used test methods, such as biopsies, provide a new test method for early and active intervention for renal fibrosis in the future, and provide new ideas and research directions for the diagnosis of fibrosis in different tissues.

There are some limitations of this study. The pathogenesis of fibrosis is extremely complex, and there are differences in renal fibrosis progression caused by different etiologies. In addition, the HYP is not an ideal parameter to define all stages of fibrosis, especially during the recovery stage [24]. Only the classical UUO model was selected for the Microprobe THz study, which may not be generalizable to the entire disease course. In subsequent studies, we will use the Microprobe THz imaging system to test different fibrosis models, as well as different biomacromolecules, which could provide more ideas for analyzing the pathogenesis of renal fibrosis, and assist in the early diagnosis and treatment of renal fibrosis in clinical practice.

## 5. Conclusions

In summary, a THz microprobe system was employed to conduct THz spectra testing of renal tissues in mice with chronic renal fibrosis. THz spatial scanning with a resolution of 20 µm was achieved, resulting in direct observation of the THz imaging spectrum to determine the spatial distribution of HYP, which is closely related to the location of fibrosis. Compared with other techniques, Microprobe THz-TDS can improve the accuracy, sensitivity, and detection speed of diagnosis. Its high sensitivity, accuracy, and speed highlight its potential in the early diagnosis, staging evaluation, and disease monitoring of biological diseases. In the future, this form of dynamic monitoring is expected to achieve real-time imaging during operation in vivo, which can greatly improve the accuracy of treatment.

## Data Availability

The data presented in this study are available on request from the corresponding author.
